# Supplementary material for: Low-frequency repetitive transcranial magnetic stimulation in patients with motor deficits after brain tumor resection: a randomized, double-blind, sham-controlled trial
Source: Front Oncol. 2024 Apr 26;14:1368924. doi: 10.3389/fonc.2024.1368924 (PMC11082392; doi:10.3389/fonc.2024.1368924)
Supplement: Supplementary file 1 [file DataSheet_1.docx]

Supplementary Material

**Low-frequency repetitive transcranial magnetic stimulation in patients with motor deficits after brain tumor resection: a randomised, double-blind, sham-controlled trial**

**Melina Engelhardt^1,2,3^*, Heike Schneider^1^, Jan Reuther^4^, Ulrike Grittner^5^, Peter Vajkoczy^1^, Thomas Picht^1,2,6^, Tizian Rosenstock^1,7^**

^1^Charité - Universitätsmedizin, corporate member of Freie Universität Berlin and Humboldt-Universität zu Berlin, Department of Neurosurgery, Charitéplatz 1, 10117 Berlin, Germany

^2^Charité – Universitätsmedizin, corporate member of Freie Universität Berlin and Humboldt-Universität zu Berlin, Einstein Center for Neurosciences, Charitéplatz 1, 10117 Berlin, Germany

³Charité – Universitätsmedizin, corporate member of Freie Universität Berlin and Humboldt-Universität zu Berlin, International Graduate Program Medical Neurosciences, Charitéplatz 1, 10117 Berlin, Germany

^4^Charité - Universitätsmedizin, corporate member of Freie Universität Berlin and Humboldt-Universität zu Berlin, Department for Physical Medicine, Charitéplatz 1, 10117 Berlin, Germany

^5^Charité - Universitätsmedizin, corporate member of Freie Universität Berlin and Humboldt-Universität zu Berlin, Institute of Biometry and Clinical Epidemiology, Charitéplatz 1, 10117 Berlin, Germany

^6^Cluster of Excellence Matters of Activity. Image Space Material, Humboldt-Universität zu Berlin, Unter den Linden 6, 10099 Berlin, Germany

^7^Berlin Institute of Health at Charité – Universitätsmedizin Berlin, BIH Biomedical Innovation Academy, BIH Charité Digital Clinician Scientist Program, Charitéplatz 1, 10117 Berlin, Germany

|  | group difference for models without interaction of group*time | p | | 7 days | p | 1 month | p | 3 months | p |
| --- | --- | --- | --- | --- | --- | --- | --- | --- | --- |
| Fugl Meyer Score, mean difference (95%CI) | -- | | | 13.78  (-9.50-37.06) | 0.351 | 18.34  (-11.40-48.04) | 0.215 | 9.47  (-19.40-38.30) | 0.505 |
| BMRC prox, OR (95%CI), ref: control | -- | | | 4.55  (0.19-109.8) | 0.351 | 2.20  (0.10-47.50) | 0.615 | 0.48  (0.02-11.10) | 0.646 |
| BMRC dist, OR (95%CI), ref: control | 13.6  (0.38-484) | | 0.152 | -- |  | -- |  | -- |  |
| Finger tapping, OR (95%CI), ref: control | 2.69  (0.24-30.8) | | 0.420 | -- |  | -- |  | -- |  |
| NIHSS, mean difference (95%CI) | -- | | | -3.95  (-7.68-0.21) | 0.039 | -4.38  (-8.12-0.64) | 0.024 | -3.83  (-7.57-0.097) | 0.045 |
| KPS, mean difference (95%CI) | -- | | | 8.34  (-16.2-32.9) | 0.488 | 17.97  (-11.8-47.7) | 0.223 | 11.84  (-17.7-41.4) | 0.415 |
| NHPT, OR (95%CI), ref: control | -- | | | 1.23  (-1.88-4.34) | 0.437 | 0.55  (-2.48-3.58) | 0.720 | 0.30  (-2.59-3.19) | 0.839 |
| EORTC, mean difference (95%CI) | -- | | | 13.0  (-15.1-41.1) | 0.354 | 12.7  (-15.8-41.1) | 0.373 | 14.9  (-15.0-44.8) | 0.320 |

**Table A.1 Impact of motor eloquent ischemia.** Treatment effects (mean group differences/odds ratios, 95% confidence intervals, p-values) for different outcomes (based on separate models for each outcome, adjusted for baseline measures and including time point, group and interaction group*time point) in the subgroup of patients with motor eloquent ischemia (n=16; 5 in rTMS group, 11 in sham group; 48 measures per model). Models for the finger tapping and distal BMRC scores did not converge when the interaction term for group * time point was included. They were therefore analysed only with a main effect for group and time point. Estimates are based on mixed models or GEEs. prox = proximal muscles, dist = distal muscles, KPS = Karnofsky Performance Status, NHPT = Nine-Hole Peg Test.

| Before imputation (complete case analysis) | | | | | | | | | | |
| --- | --- | --- | --- | --- | --- | --- | --- | --- | --- | --- |
|  | **N individuals** | **N measures** | **group difference for models without interaction of group*time** | **p** | **7 days** | **p** | **1 month** | **p** | **3 months** | **p** |
| Fugl Meyer Score, mean difference (95%CI) | 27 | 52 | -- | | 5.78  (-7.43-19.00) | 0.378 | 15.22  (-1.40-31.83) | 0.072 | -2.33 (-19.66-15.00) | 0.788 |
| BMRC prox, OR (95%CI), ref: control | 29 | 71 | -- | | 1.65  (0.18-15.23) | 0.658 | 3.19  (0.23-45.53) | 0.384 | 0.15  (0.01-2.35) | 0.175 |
| BMRC dist, OR (95%CI), ref: control | 30 | 71 | -- | | 8.62  (0.07-1078.83) | 0.382 | 511.00  (1.55-168746.16) | 0.035 | 0.42  (0.002-80.65) | 0.747 |
| Finger tapping, OR (95%CI), ref: control | 27 | 54 | 6.29 (0.94-42.00) | 0.058 | -- |  | **--** |  | **--** |  |
| NIHSS, mean difference (95%CI) | 29 | 70 | -- | | -2.09 (-4.21-0.02) | 0.053 | -1.91 (-4.21-0.39) | 0.101 | -0.57 (-2.89-1.74) | 0.621 |
| KPS, mean difference (95%CI) | 30 | 71 | **--** | | 2.58 (-8.26-13.41) | 0.633 | 9.15 (-2.81-21.10) | 0.131 | -0.46 (-12.55-11.64) | 0.902 |
| NHPT, OR (95%CI), ref: control | 30 | 66 | -- | | 1.43  (0.22-9.26) | 0.708 | 1.88 (0.20-17.27) | 0.579 | 1.00 (0.10-10.17) | 1.000 |
| EORTC QoL, mean difference (95%CI) | 21 | 47 | **--** | | 2.19 (-11.60-15.92) | 0.748 | 4.42 (-12.70-21.58) | 0.605 | 3.10 (-13.50-19.67) | 0.707 |
| After single imputation of informative missings | | | | | | | | | | |
| Fugl Meyer Score, mean difference (95%CI) | 27 | 59 | -- | | 5.50 (-9.37-20.37) | 0.457 | 7.44 (-10.12-25.00) | 0.398 | -4.23 (-22.09-13.60) | 0.636 |
| BMRC prox, OR (95%CI), ref: control | 30 | 79 | -- | | 1.51 (0.12-19.20) | 0.752 | 2.65 (0.18-39.70) | 0.481 | 0.21 (0.01-3.20) | 0.258 |
| BMRC dist, OR (95%CI), ref: control | 30 | 79 | -- | | 4.90 (0.10-234.00) | 0.424 | 46.90 (0.55-4030.00) | 0.090 | 0.20 (0.01-12.00) | 0.464 |
| Finger tapping, OR (95%CI), ref: control | 28 | 61 | 3.86 (0.59-25.10) | 0.158 | -- |  | -- |  | -- |  |
| NIHSS, mean difference (95%CI) | 29 | 78 | -- | | -2.08 (-4.68-0.52) | 0.114 | -1.73 (-4.44-0.99) | 0.207 | -7.13 (-24.71-10.44) | 0.419 |
| KPS, mean difference (95%CI) | 30 | 79 | -- | | 1.12 (-15.30-17.53) | 0.891 | 3.84  (-13.88-21.55) | 0.666 | -0.46  (-12.55-11.64) | 0.940 |
| NHPT, OR (95%CI), ref: control | 30 | 75 | -- | | 1.43  (0.22-9.26) | 0.708 | 0.90 (0.13-6.08) | 0.914 | 0.58 (0.08-4.39) | 0.601 |
| EORTC, mean difference (95%CI) | 23 | 56 | -- | | 6.41 (-11.60-24.44) | 0.476 | 4.89 (-15.60-25.41) | 0.633 | -4.69  (-24.00-14.59) | 0.626 |

**Table A.2 Sensitivity analyses.** Treatment effects (mean group differences/odds ratios, 95% confidence intervals, p-values) for different outcomes before imputation and after single imputation for informative missings (based on separate models for each outcome, adjusted for baseline measures and including time point, group and interaction group*time point). Models for the finger tapping did not converge when the interaction term for group * time point was included. They were therefore analysed only with a main effect for group and timepoint. Estimates are based on mixed models or GEEs. prox = proximal muscles, dist = distal muscles, KPS = Karnofsky Performance Status, NHPT = Nine-Hole Peg Test.
